# Supplementary material for: Inner southern magnetosphere observation of Mercury via SERENA ion sensors in BepiColombo mission
Source: Nat Commun. 2022 Nov 30;13:7390. doi: 10.1038/s41467-022-34988-x (PMC9712576; doi:10.1038/s41467-022-34988-x)
Supplement: Supplementary file 1 — Supplementary Information [file 41467_2022_34988_MOESM1_ESM.pdf]

Inner southern magnetosphere observation of Mercury via SERENA ion sensors in BepiColombo mission

S. Orsini[1*], et al.

1. Institute of Space Astrophysics and Planetology, INAF, Rome, Italy.
*Corresponding author: stefano.orsini@inaf.it

Supplementary Information

Methods

- Data Downlink and processing data pipeline

Payload telemetry (P/L TM) data collected by BC is downlinked to Earth through the Estrack ground stations antennas of ESA (https://www.esa.int/Enabling_Support/Operations/ESA_Ground_Stations/Estrack_ground_stations) and the Deep Space Network (DSN) antennas of NASA Ground Segment (https://www.nasa.gov/directorates/heo/scan/services/networks/deep_space_network/about). From there, data are transferred and stored at the Ground Operation System's Data Dissemination System (EDDS) server located at ESA-ESOC (Germany) where they are ready to be retrieved directly by the instrument teams or by the ESA Science Ground Segment (SGS) servers located ad ESA-ESAC (Spain). P/L TM is binary data produced according to the SCOS-2000 standard (https://www.esa.int/Enabling_Support/Operations/Ground_Systems_Engineering/SCOS-2000). They basically consist of science (SC) measurements and instrument housekeeping (HK) data. Once received on-ground, TM data are sorted by generation time (Spacecraft (S/C) Elapsed Time -SCET-), packed and stored into the EDDS server, and finally provided to the instrument teams and SGS team as DDS binary data. SERENA team has developed a scientific data processing and archiving architecture in cooperation with BC SGS team. The SERENA instrument pipeline is developed by the SERENA team. As for any other BepiColombo Instrument (e.g: 1), it basically consists of two main routines: telemetry-to-raw (TM2RAW) and raw-to-calibrated (RAW2CAL), generating all HK and SC instrument data, and in some additional sub-routines generating ancillary data (e.g. documentation, geometry files, ...). Only the TM2RAW routine has been implemented into the pipeline so far, while RAW2CAL routine is under development and will be implemented soon. For this reason, a reference to this work is still not available, but an article will be issued as soon as the pipeline is completed.The pipeline is designed to run at both INAF-IAPS and SGS, and provides validated data sets and a quick-look analysis tool. Data processed by the pipeline are formatted according to the Planetary Data System, Version 4 standards (PDS4, https://pds.nasa.gov/datastandards/about/ and https://www.cosmos.esa.int/web/psa/pds4-standards), maintained by NASA, and archived into the public Planetary Science Archive (PSA, https://www.cosmos.esa.int/web/psa/psa-introduction), maintained by ESA. The pipeline has two different processing branches: telemetry-to-raw (TM2RAW) data and raw-to-calibrated (RAW2CAL) data. The former generates RAW data from the TM files, the latter uses RAW data to produce CALibrated data. Usually, the two modules work in sequence, but they can also work separately, e.g. for instrument health checks (TM2RAW) or re-calibration purposes (RAW2CAL). The inputs of TM2RAW are the DDS files retrieved by the S/C and the SPICE kernels provided by the NASA's Navigation and Ancillary Information Facility (NAIF) team (https://naif.jpl.nasa.gov/naif/spiceconcept.html). The TM2RAW routine extracts all relevant binary data packets (SC and HK) from DDS files, generates proper UTC time stamps, and converts the data into ASCII RAW data files. The SPICE kernels contain the information to calculate the spacecraft position, the FoV of the sensors and the parameters for the time conversions for each SC and HK packet. Additionally, PDS4 label files in XML format are generated to provide ancillary information. The RAW data includes unprocessed (ADC counts) original SC and HK data from the instrument which is mainly used for diagnostic purposes. If compression, reformatting, packetization, or other translation has been applied to facilitate data transmission or storage, all data generated by the pipeline are available as ASCII files and grouped in RAW, CALIBRATED & DERIVED data. Below this level, data are grouped by mission phases, e.g.: Near Earth Commissioning or Cruise. The next logical level distinguishes data sensor by sensor, and then separates the housekeeping HK from science SC data. Additionally, browse plots for a quick data

overview and useful information for the data end-user can be found in the archived datasets. In the present paper only RAW data are shown.

- PICAM (Planetary Ion CAMera) ion mass spectrometer)

PICAM is an all-sky camera (1, 3) capable of measuring the 3D velocity distribution of ions up to ~3 keV energies, and their mass spectrum extending up to ~132 amu (Xenon). SERENA-PICAM is an omnidirectional sensor with no angular scanning, hence having a very efficient duty cycle. A photo of the flight hardware is illustrated in Suplementary Figure 1, and its specification, such as field of view (FoV), energy range, geometrical factor and mass resolution are represented in Suplementary Table 1. More details regarding the instrument design, operational modes, calibration tests and science objectives are explained in (3). During BepiColombo first flyby at Mercury, SERENA-PICAM was chosen to operate in two major modes, Image (IMG) and Time of Flight (ToF). The IMG mode provided ion counts for 32 energy channels, and 31 anodes The IMG mode was operated in Solar Wind covering 460 to 2100 eV, and at Bowshock from 11 to 2735 eV. Near the planet, SERENA-PICAM switched to ToF mode with the latter energy range, but a high mass resolution to look at the planetary ion species. The boresight vector of PICAM was perpendicular to the Sun-MPO line throughout the flyby. The details of each mode are also shown in Suplementary Table 1.

- MIPA (Miniature Ion Precipitation Analyzer)

MIPA (Suplementary Figure 2) is a highly optimized compact (~600 g) ion mass analyzer of the SWIP-family (4, 3) capable of measuring the 3D velocity distribution of ions in the energy range 10 eV – 15 keV with mass resolution to resolve the main plasma species (1,2,4,8,> 16 amu). MIPA is a single pixel fast sweeping sensor (8 ms sampling time per energy level) capable of covering 96 energies x 24 directions for just 18 sec (full mode). MIPA uses ion – surface interaction combined with the time-of-flight (TOF) analysis of the secondary electrons for the ion mass determination. MIPA does not have its own data processing and is controlled by the SERENA processing unit. Suplementary Table 2 sums up the basic performances. During the time interval used in this analysis MIPA operated in the full angular coverage mode (24 viewing pixels) for 32 energy steps (3 steps integrated) with fastest time resolution (20 sec /3d, 18 sec + 2 sec idle, no time integration). 8 TOF bins were used which is sufficient to resolve hydrogen and heavy ions ($\geq$ 2 amu)

References

1. Heyner, D., et al.: The BepiColombo Planetary Magnetometer MPO-MAG: what can we learn from the Hermean Magnetic Field?. Space Science Reviews (2021) 217:52, https://doi.org/10.1007/s11214-021-00822-x

2. Vaisberg, O., et al.: 'The 2π charged particles analyzer: all-sky camera concept and development for space missions'. *J Geophys Res*, 121(12), 11750–11765 (2001). https://doi.org/10.1002/2016JA022568.

3. Orsini, S., et al.: 'SERENA: Particle Instrument Suite for Determining the Sun-Mercury Interaction from BepiColombo'. *Space Sci Rev*, 217, 11 (2021). https://doi.org/10.1007/s11214-020-00787-3.

4. Wieser, M., S. Barabash: 'A family for miniature, easily reconfigurable particle sensors for space plasma measurements', *J Geophys Res* (2016). https://doi.org/ 10.1002/2016JA022799.

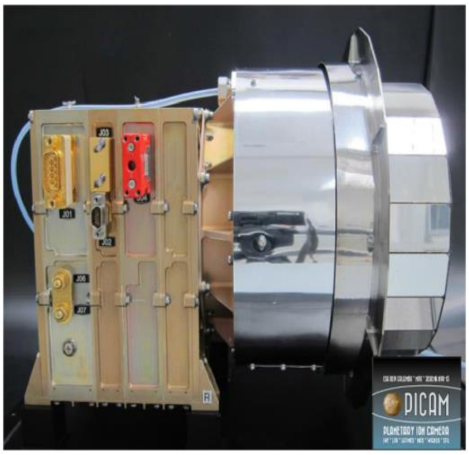

Supplementary Figure 1. **Side view of the PICAM flight model**

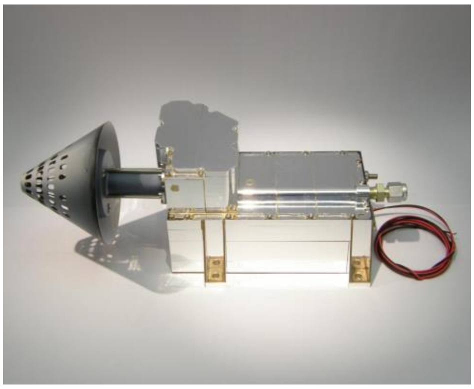

Supplementary Figure 2. **MIPA flight model.** The aperture is protected by the conic radiator with entrance holes. The hot section (radiator and angular sweeping electrodes inside) operates at 400°C and is decoupled from the rest of the instrument by a titanium decoupling tube.

**Energy range**	~10 - 3000 eV
**Energy resolution** (ΔE/E)	<15%
**Viewing angle**	3D, 1.5π
**Angular resolution**	~20° x 60°
**Mass range**	1-132 AMU
**Mass resolution** (M/ΔM)	> 50
**Time resolution**	<1 m
**Sampling time**	1-320 s
**Effective Geo. factor** (S Ω ΔE/E)	10^{-4}– 10^{-6} (cm^2 sr eV)/eV

**Run time** [minute]	995	80	90
**Science region**	Solar Wind	Magnetosphere	Bowshock, Solar Wind
**PICAM mode**	IMG 4	ToF 11 (S)	IMG 4
**No. of anodes**	31	4	31
**ToF bins**	NA	512 bins	NA
**Energies measured**	460-2100 eV (32 ch.)	11-2735 eV (32 ch.)	11-2735 eV (32 ch.)
**Temporal resolution** [s]	64	128	64

Supplementary Table 1. **PICAM specifications**. The left half represents the general specifications for SERENA-PICAM, and the right half the operational details of SERENA-PICAM during the first Mercury Flyby.

Parameter	Value
Energy range	10 eV – 15 keV
Energy resolution $\Delta E/E$	7.3%
Viewing angle	90° x 180°
Angular resolution (varibale)	40°x20° (max pixel)<br>5° x 15 (min pixel)
Mass range, amu	1 - 50
Mass resolution, $M/\Delta M$	2-5
Time resolution, sec	18, Full Angular– Energy cycle (24A x 96E)
Efficiency, ε	12%
Geometrical factor, w/o ε, maximum possible base line	24 x $2\cdot 10^6$ cm^2 sr eV/eV
Dynamical range	$2\cdot 10^7$

Supplementary Table 2. **MIPA basic performances**
